# Does the shoe really fit? Characterising ill-fitting footwear among community-dwelling older adults attending geriatric services: an observational cross-sectional study

**DOI:** 10.1186/s12877-020-1448-9

**Published:** 2020-02-13

**Authors:** B. O’Rourke, M. E. Walsh, R. Brophy, S. Vallely, N. Murphy, B. Conroy, C. Cunningham, N. F. Horgan

**Affiliations:** 1 School of Physiotherapy, Royal College of Surgeons in Ireland, Dublin 2, Ireland; 2 Health Research Board (HRB) Centre for Primary Care Research, Royal College of Surgeons in Ireland, Dublin 2, Ireland; 3 Physiotherapy Department, St James’s hospital, Dublin 8, Ireland; 4 Robert Mayne Day Hospital, St James’s hospital, Dublin 8, Ireland; 5 Mercers Institute for Successful Ageing (MISA), St James’s hospital, Dublin 8, Ireland

**Keywords:** Shoes, Footwear fit, Mobility, Accidental falls

## Abstract

**Background:**

Falls in older people are common and can result in loss of confidence, fear of falling, restriction in activity and loss of independence. Causes of falls are multi-factorial. There is a paucity of research assessing the footwear characteristics among older people who are at high risk of falls, internationally and in the Irish setting. The aim of this study was to examine the proportion of older adults attending a geriatric day hospital in Ireland who were wearing incorrectly sized shoes.

**Methods:**

A consecutive sample of 111 older adults aged 60 years and over attending a geriatric day hospital in a large Irish teaching hospital was recruited. Demographic data including age, mobility, medications, co-habitation status, footwear worn at home and falls history were recorded. Shoe size and foot length were measured in millimetres using an internal shoe gauge and SATRA shoe size stick, respectively. Participants’ self-reported shoe size was recorded. Footwear was assessed using the Footwear Assessment Form (FAF). A Timed Up and Go (TUG) score was recorded. Functional independence was assessed using the Nottingham Extended Activities of Daily Living (NEADL) Scale. The primary outcome of interest in this study was selected as having footwear within the suggested range (10 to 15 mm) on at least one foot. Participants who met this definition were compared to those with ill-fitting footwear on both feet using Chi-square tests, T-tests or Mann–Whitney U tests.

**Results:**

The mean difference between shoe length and foot length was 18.6 mm (SD: 9.6 mm). Overall, 72% of participants were wearing footwear that did not fit correctly on both feet, 90% had shoes with smooth, partly worn or fully worn sole treading and 67% reported wearing slippers at home. Participant age, TUG score and NEADL score were not associated with ill-fitting footwear.

**Conclusions:**

Wearing incorrectly fitting shoes and shoes with unsafe features was common among older adults attending geriatric day services in this study. A large number of participants reported wearing slippers at home.

## Key points

- It is common among older adults attending a day hospital to wear ill-fitting shoes
- A large proportion of older adults report wearing slippers in their own homes
- There is need for interventions for older adults specific to footwear advice, education and remediation

## Background

Over 7000 older adults are admitted to Irish hospitals with a fall annually [1]. Falls and fall-related fractures are a major risk to older people as well as being a huge cost to society [2], with nearly one in three community-dwelling older adults falling annually [3]. Falls can cause serious injury resulting in disability, nursing home admission and death [4]. Even when there is no serious injury, falls impact on the individual, resulting in loss of confidence, fear of falling, restriction in activity and a decreased quality of life [5]. The causes of falls in older people are multifactorial, including poor lower limb proprioception, visual impairment, side-effects of psychoactive medications, decreased reaction time and decreased lower limb muscle strength [6, 7]. In addition to these intrinsic factors, it has been suggested that falls may also be caused by extrinsic factors such as environmental hazards and unsafe footwear [8, 9].

Inappropriate footwear refers to both particular types of footwear with unsafe features, as described in the Footwear Assessment Form (FAF) [10], and also footwear of an incorrect size [11]. Wearing inappropriate footwear has been associated with falls [12]. Over half of the older participants in the MOBILIZE Boston Study were wearing slippers, barefoot or socks without shoes when they experienced an in-home fall [11]. A recent systematic review found inadequate evidence linking particular footwear styles with falls among healthy community dwelling older adults [13], however, trip-related falls may be linked to wearing slippers or ill-fitting shoes without proper fixation in an older population [14].

A shoe’s material and tread design can affect the coefficient of friction on the walking surface, which may influence the risk of slipping [15, 16]. Thin, hard-soled footwear with high collars are advised to reduce the risk of falling [17, 18]. Heel height and width may affect a shoe’s tendency to tip sideways on an uneven surface, as well as gait and posture [6, 19]. High-heeled shoes have been linked to impaired balance in older people [14, 20]. The characteristics of footwear among older people who attend geriatric services and may be frail with co-morbidities and falls-risk have not been described.

The aim of this study was to examine the proportion of older individuals attending a geriatric day hospital who were wearing incorrectly sized shoes. A secondary aim was to determine the characteristics of footwear worn by this population.

## Methods

### Design

This was an observational cross-sectional study. The authors followed the STROBE guidelines for the reporting of observational studies [21].

#### Participants

A consecutive convenience sample of older individuals presenting as outpatients to a geriatric day hospital of an acute general teaching hospital were invited to participate in the study. Recruitment took place between June–July 2017. Participants were included if they were aged over 60 years, were living at home, were attending the geriatric day hospital, agreed to participate in the study, were able to stand independently and had the ability to understand simple instructions so as to allow completion of assessments. The senior physiotherapist in the day hospital acted as a gatekeeper. Participants deemed to be eligible were invited to take part in the study and provided with an information leaflet, and gave written informed consent. The study was approved by the hospital’s Research Ethics Committee (REC2017-05-6).

### Procedure

#### Participant characteristics

Information on age, level of mobility, and co-habitation status (living alone or with others) was determined from participant self-report and medications were recorded from participant medical records. The Nottingham Extended Activities of Daily Living (NEADL) Scale was conducted with each participant to assess their self-reported level of functional independence and scored from 0 (low independence) to 22 (high independence) [22]. Functional mobility, as measured by a recent ‘Timed Up and Go’ (TUG) score was retrieved from the physiotherapy department records [23]. Polypharmacy, a known risk factor for falls, was defined as five or more medications daily [24, 25]. Self reported falls-history (one-month and six-month) was also collected [26]. The definition of a fall was taken from the work of Tinetti [27]. Participant functional ability was characterised in this study by use of mobility aid, TUG score and NEADL score.

#### Fit of footwear

The definition used in this study for correctly fitting shoe size was taken from the work by Chantelau and Gede [28]. They describe the need for a gap of 10 to 15 mm between the toes and the anterior of the shoe to allow “extra space for the toes when extending during walking and standing” [28]. A similar measure of “approximately 1.5cm between the hallux and the shoe end” was used by Menant et al. [14]. The primary outcome of interest in this study was selected as having footwear within the suggested range (10 to 15 mm) on at least one foot. This was decided as discrepancy between right and left sizes has been found to be one of the most common reasons for recommendation of larger shoes among older adults, as split-sizes would need custom order [29]. Foot and footwear assessments were administered in a quiet, bright physiotherapy treatment room by a student physiotherapist who had received specific training. We piloted the research procedures in the first week of the study to ensure standardised foot and footwear measurement.

#### Foot measurements

**Fig. 1:**
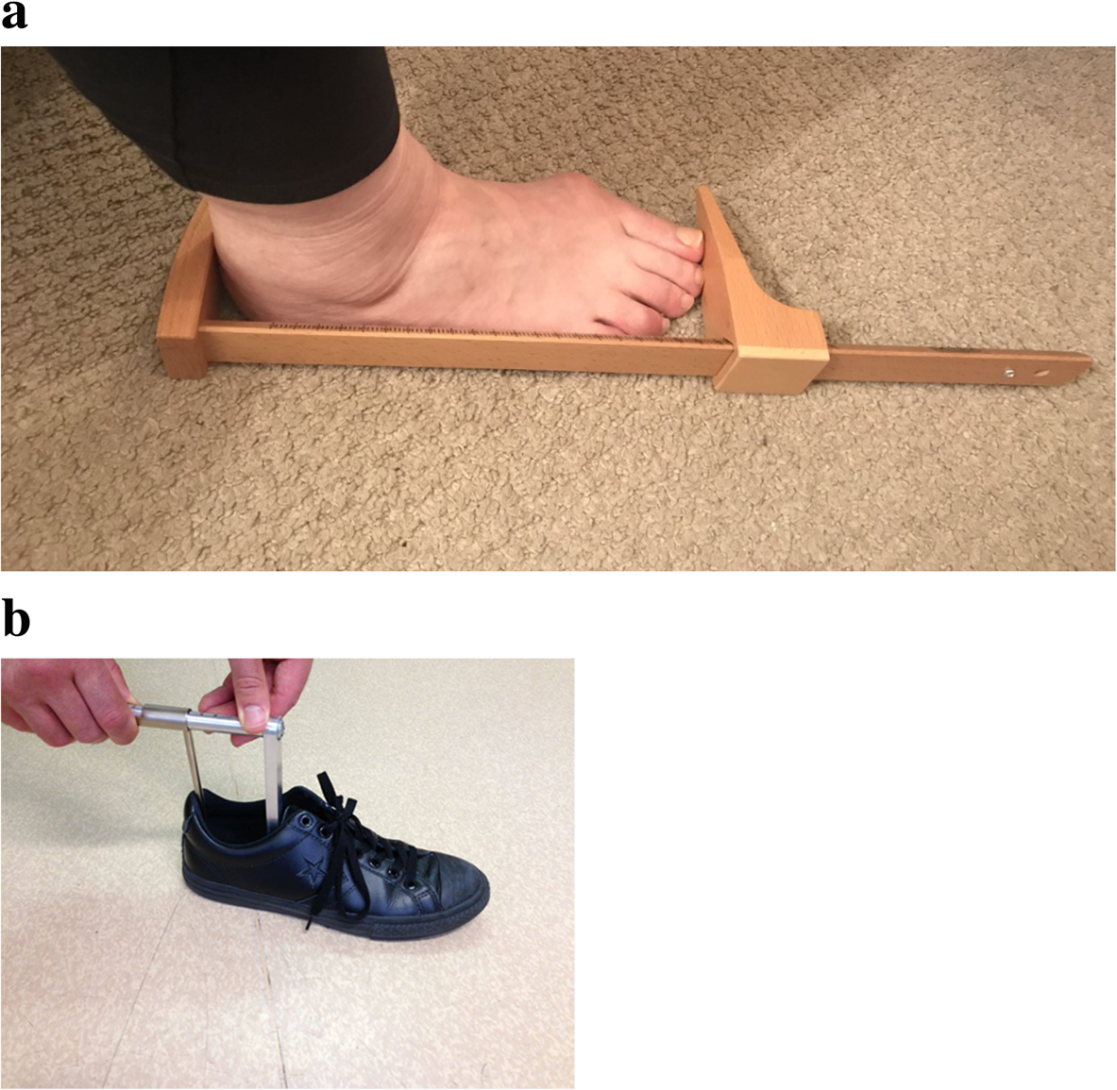
**a**: SATRA shoe size stick. **b** Internal Shoe Size Gauge

Both feet of each participant were measured in millimetres using a SATRA shoe size stick. Each participant stood barefoot and relaxed, with the feet slightly apart and with the weight evenly distributed between both feet. The fixed anvil of the SATRA shoe size stick (Fig. 1a) was placed behind the heel of the foot (which was barefoot, socks off) being measured with the researcher firmly holding the participant’s ankle and device together. The researcher then moved the sliding caliper up to the longest toe and noted the foot length indicated. It is important to note that the longest toe was not necessarily the first toe. The same procedure was repeated for the other foot. The participant’s self-reported shoe size was also recorded.

#### Footwear measurements

The participant’s footwear was placed on a firm level surface. A calibrated Internal Shoe Size Gauge® (SATRA, UK) was then placed into the shoe and the flat bar of the device pushed into the shoe until it clearly contacted the end of the toe box (Fig. 1b). The slide of the device was then adjusted until the rear curved bar section touched the heel of the shoe. The internal length of the shoe was recorded in mm. The same procedure was then repeated for the other shoe.

#### Footwear assessment

Footwear was assessed using the Footwear Assessment Form (FAF), which is a reliable tool for the assessment of shoe style, heel height, fixation, heel counter stiffness, longitudinal sole rigidity, sole flexion point, tread pattern and sole hardness [10]. We asked all participants if the shoes worn at the time of the assessments were their regular daily footwear.

### Statistical analyses

The difference between foot length and internal shoe-length was calculated in mm. The proportion (with 95% confidence intervals) of participants whose foot to shoe length difference was outside the 10 to 15 mm range was calculated. Those with shoes fitting on at least one foot were compared to those with ill-fitting footwear on both feet using the Chi-square test for categorical variables and the T-test or Mann–Whitney U test for continuous variables depending on normal quantile plots. Statistical analyses were carried out using SPSS® Statistics Version 16.

## Results

### Participant characteristics

A total of 133 participants were screened, 2 declined to participate and 111 participants were assessed. There were 44 males and 67 females assessed with a mean age of 81.6 years (range 63–99, SD = 7.5). The mean NEADL score was 12.7 (SD = 4.6). The median TUG score, available for 92 participants (83%), was 20 s (IQR = 16–25.7). A walking aid was used by 80% (*n* = 89) of participants, 43% (*n* = 48) reported living alone. Over two-thirds (67% (*n* = 74)) reported wearing slippers at home. Close to half of participants (51% (*n* = 57)) reported having fallen in the last 6 months. A large proportion (87% (*n* = 96)) were taking five or more medications.

### Footwear characteristics

Table 1 describes the characteristics of shoes worn by the participants. The median UK shoe size for males was a size 9 and for females it was a size 6. On the day of assessment the majority, 59% of men (*n* = 44) and 46% women (*n* = 67), wore a walking style shoe. Table 1 shows the distribution of other shoe types by gender.

**Table 1:** Characteristics of shoes worn by participants

	Male (*n* = 44)	Female (*n* = 67)	Total (*n* = 111)
	Right (SD): Left (SD)	Right (SD): Left (SD)	Right (SD): Left (SD)
Mean Foot Length mm	264.3 (18.3): 264.3 (18.9)	236.9 (18.3): 236.3 (19)	247.7 (18.3): 247.4 (18.9)
Mean Internal Shoe Size mm	283 (19.2): 283 (19.2)	255.1 (19.3): 255.2 (19.2)	266.1 (19.2): 266.2 (19.1)
Mean difference between foot and shoe length	18.8 (10): 18.7 (10.4)	18.6 (9.95): 19.4 (10.3)	18.4 (9.9): 18.8 (10.3)
Shoe Characteristics (FAF):	n (%)	n (%)	n (%)
Shoes Fitting on both feet	3 (7)	4 (6)	7 (6)
Shoes Fitting on at least one foot	14 (32)	17 (25)	31 (28)
Shoes with no fixation	8 (18)	21 (31)	29 (26)
Heel Counter Stiffness > 45	25 (57)	45 (67)	70 (63)
Longitudinal Sole Rigidity > 45	34 (77)	56 (85)	90 (81)
Sole Flexion Point not at MTPJs	3 (7)	5 (7)	8 (7)
Smooth, partly worn or fully worn sole	42 (95)	58 (87)	100 (90)
Shoe Type (FAF):			
Walking Shoes	26 (23)	31 (28)	57 (51)
Sandals	3 (3)	14 (13)	17 (15)
Athletic Shoes	4 (4)	6 (5)	10 (9)
Moccasins	2 (2)	6 (5)	8 (7)
Court Shoes	0 (0)	3 (3)	3 (3)
Oxford Shoes	5 (5)	1 (1)	6 (5)
Slippers	2 (2)	3 (3)	5 (5)
Other	2 (2)	3 (3)	5 (5)

Shoe Characteristics and Shoe Type as described in the Footwear Assessment Form (FAF)

Two-thirds of participants (67%, *n* = 74) reported that they wore slippers at home (for comfort and ease of putting on/taking off). According to the Footwear Assessment Form (FAF) no participants’ shoes had 5 or more unsafe features, however, there were several features associated with unsafe shoes noted: 26% (*n* = 29) had shoes with no fixation, 63% (*n* = 70) had shoes with a heel counter stiffness > 45°, 81% had shoes with a longitudinal sole rigidity > 45° and 90% (*n* = 100) of participants had smooth, partly worn or fully worn sole treading on their shoes. A third of participants (34%), had a difference between their self reported shoe size and the actual size of shoes they were wearing.

**Fig. 2:**
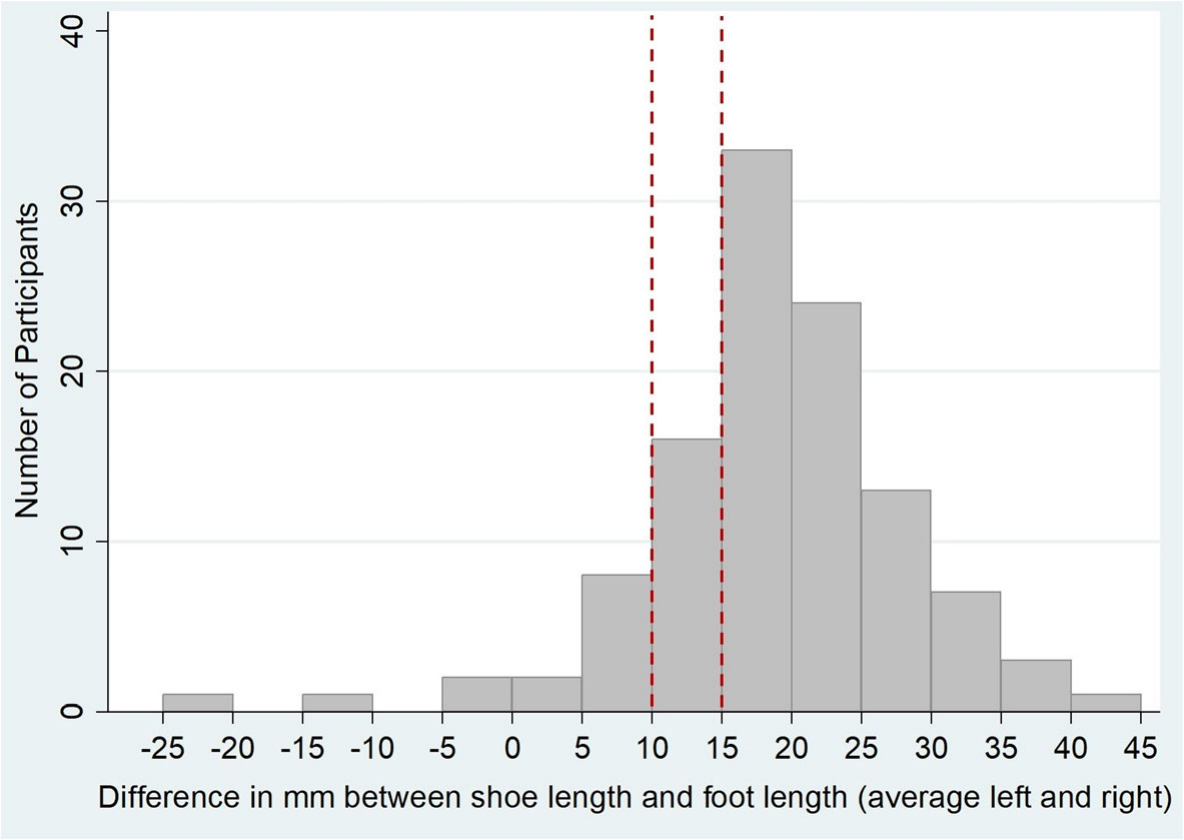
Distribution of discrepancy between foot length and shoe length: the dashed line shows the recommended range of 10-15 mm difference

The mean difference between shoe length and foot length was 18.6 mm (SD: 9.6 mm). Figure 2 shows the distribution of the average difference and Table 1 shows this difference broken down by gender and side of foot. Only 6% of participants (*n* = 7) (95% CI 2–11%) were defined as having shoes correctly fitting on both feet, i.e. 10-15 mm difference between shoe length and foot length. Correctly fitting shoes on at least one foot was identified in 28% (*n* = 31) of participants (95% CI 20–36%). Table 2 compares participants who were wearing correctly fitting shoes on at least one foot and ill-fitting shoes. Differences in demographics, functional mobility, functional independence and falls history between the two groups were small and none reached statistical significance. In total, 56% (*n* = 45) of those with ill-fitting footwear fell in the last six months compared to 39% (*n* = 12) of those with one shoe within recommend range (*p* = 0.09).

**Table 2:** Comparison of Participants with Correctly fitting and Ill-fitting shoes

	*Shoes Fitting on at least one foot n = 31* *n (%)*	*Shoes Not Fitting on both feet n = 80* *n (%)*	*Test*	*P-value*
*Female*	17 (55)	50 (62.5)	Chi^2^	0.46
*Using Walking Aid*	23 (74)	66 (83)	Chi^2^	0.33
*Falls in last 6 months*	12 (39)	45 (56)	Chi^2^	0.09
*Falls in last 1 month*	5 (16)	19 (24)	Chi^2^	0.38
*Co-habitation status (% living alone)*	12 (39)	36 (45)	Chi^2^	0.55
*Mean Age (SD) years*	82.2 (±7.4)	81.4 (±7.5)	T-Test	0.63
*NEADL Mean Score out of 22. (SD)*	13.1 (±4.8)	12.6 (±4.5)	T-Test	0.57
*TUG Median Score seconds (IQR)*	20 (16–25.7)^a^	20 (14.5–30)^b^	Mann-Whitney U Test	0.20

NEADL: Nottingham Extended Activities of Daily Living

^a^based on TUG scores obtained of 25 out of 31 participants

^b^ based on TUG scores obtained of 67 out of 80 participants

## Discussion

### Summary of findings in context of other literature

To the knowledge of the authors, this is the largest study to date to estimate the proportion of older adults attending a geriatric day hospital who were wearing inappropriate shoes. Most participants were wearing footwear that could be described as inappropriate as it was either ill-fitting or had unsafe features. We found a high number of older people to be wearing footwear larger than recommended on both feet. This is consistent with previous studies in older populations [30–33]. This may put these individuals at risk of trip-related falls [14]. Furthermore, the majority of participants were wearing footwear with several unsafe features including shoes with a heel counter stiffness greater than 45°, shoes with a longitudinal sole rigidity greater than 45° and shoes that had smooth, partly worn or fully worn sole tread pattern. As expected, this sample of participants represents a group with functional limitations, as 80% use walking aids and 87% take at least five or more medications daily. The mean NEADL score of 12.7 showed relatively low levels of independence in instrumental ADLs compared to older adults living in the community [34]. Furthermore, the median time of 20 s to complete the TUG has previously been found to be associated with a high level of frailty in older community-dwelling adults in Ireland [35]. The very high rate of ill-fitting footwear identified in this population is striking both because they are a group that would be considered at high risk of falls and other adverse events, and also because they are a group that has contact with healthcare professionals and should be in receipt of advice from doctors, physiotherapists and occupational therapists. While 56% of those with ill-fitting footwear fell in the last six months compared to 39% of those with one shoe within recommend range, this was not found to be significantly different with the current sample size. As a smaller number than expected wore correctly fitting shoes based on our definition (*n* = 30), the study was likely underpowered to detect a difference in actual fall events.

Previous footwear studies have identified the difficulty in convincing older adults to purchase appropriate footwear through educational interventions [36]. However, advice and education from health professionals as a behavioural change intervention has been shown to be effective in other healthcare issues such as smoking cessation [37]. Further research into current footwear education interventions being provided to older adults within and outside geriatric services is warranted. The footwear patterns and level of awareness of footwear are unknown among the broader population of community-dwelling older adults in Ireland who do not attend geriatric services. To the knowledge of the authors there are no recent studies investigating footwear patterns and level of awareness of footwear among this population and this is an area for further research.

Previous literature has suggested that podiatry and footwear interventions may prevent falls [38, 39]. The finding in our study that the majority of older adults attending geriatric services wore ill-fitting footwear suggests that podiatric assessment may be warranted in this setting.

The large number of participants who reported wearing slippers at home was an important finding as slippers may be a risk for falls in an older population [11, 12, 14]. The main reasons for selection of footwear for indoor use by older adults have been identified as comfort and low cost, and indoor footwear is also rarely replaced [36]. This method for choosing footwear appears to be consistent with the findings in this study and may contribute to risks of falling for older adults at home. Advice regarding safe and appropriate footwear may be a useful intervention to address this risk and an area for further research.

### Strengths and limitations of the study

To the knowledge of the authors, this is the first study to estimate the proportion of older adults attending a geriatric day hospital who were wearing inappropriate shoes. This is a descriptive cross-sectional study of footwear worn by a consecutive convenience sample of older adults who are community-dwelling but who have been referred to a geriatric day hospital for social, rehabilitative and medical assessment and treatment. It provides a detailed description of common potentially problematic footwear characteristics in this group. It may not be generalisable to all community-dwelling older adults but may be relevant in clinical assessment and treatment settings. Furthermore, the sample size of 111 participants is relatively small, leading to a wide 95% confidence interval around the estimate proportion wearing ill-fitting footwear. We did not measure the width of participant’s shoes or feet during this study and thus we are unable to determine whether participants were wearing shoes that were too narrow or too wide for their feet. Not measuring the width of the feet of the participants is a major limitation of this study, particularly considering that the feet tend to become wider and flatter with the ageing process [40]. We were unable to assess the shoes or slippers worn at home by the participants, as we only assessed the shoes that were worn into the day hospital on the day of assessment. In addition, it was not identified if the shoes that were examined in the study were worn when these participants had falls. The use of self-report measures is also a limitation.

### Implications for future research and practice

Older adults in Ireland who are aged over 65 years make up just over 12% of the population and this number is expected to increase in coming years [41]. The wearing of incorrectly fitting shoes is also strongly associated with pathology of the forefoot and with foot pain [31, 33]. The assessment of such conditions was outside the scope of this study but is an area that should be explored in future research. There is a need for a routine uncomplicated assessment of footwear for older adults, which could help to prevent foot disorders and possibly reduce the risk of falls.

## Conclusion

Wearing incorrectly fitting shoes and shoes with unsafe features was common among older adults attending geriatric day services in this study. Wearing slippers at home also seems to be a common occurrence among this population. These findings would offer benefit to future participants in relation to addressing foot pathology and the need for interventions specific to footwear advice, education and remediation.

## Data Availability

The datasets used and/or analysed during the current study are available from the corresponding author on reasonable request.

## Abbreviations

FAF: Footwear Assessment Form
NEADL: Nottingham Extended Activities of Daily Living Scale
TUG: Timed Up and Go
